# 4-Ferrocenylphenol

**DOI:** 10.1107/S1600536808039524

**Published:** 2008-11-29

**Authors:** Vincent O. Nyamori, Muhammad D. Bala

**Affiliations:** a School of Chemistry, University of KwaZulu-Natal, Westville Campus, Private Bag X54001, Durban 4000, South Africa

## Abstract

The title compound, [Fe(C_5_H_5_)(C_11_H_9_O)], is of inter­est as a precursor to the synthesis of cheap ferrocene-based liquid crystals. The –OH substituent only results in weak C—H⋯O weak inter­actions between one of cyclo­penta­dienyl (Cp) ring H atoms and the O atom of a neighbouring mol­ecule with a distance of 3.308 (3) Å between the donor and acceptor atoms. The inter­planar angle between the Cp and benzene rings is 13.0 (4)°. There are also weak O—H⋯π and C—H⋯π inter­actions involving the unsubstituted Cp and the benzene ring, respectively.

## Related literature

For general background, see: Togni & Hayashi (1995 ▶); Imrie *et al.* (2002 ▶). For related structures, see: Imrie *et al.* (2003 ▶); Nyamori & Bala (2008*a* ▶,*b* ▶). For related syntheses, see: Guillaneux & Kagan (1995 ▶); Foxman & Rosenblum (1993 ▶); Tsukazaki *et al.* (1996 ▶); Lin *et al.* (1995 ▶); Knapp & Rehahn, (1993 ▶).
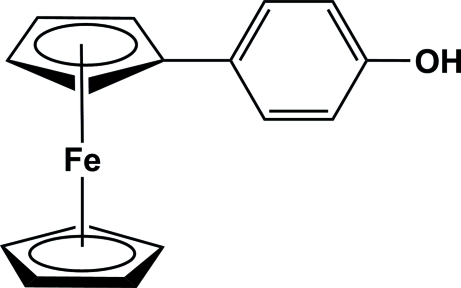


## Experimental

### 

#### Crystal data

[Fe(C_5_H_5_)(C_11_H_9_O)]
*M* *_r_* = 278.12
Orthorhombic, 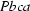

*a* = 9.950 (2) Å
*b* = 7.9205 (17) Å
*c* = 31.046 (6) Å
*V* = 2446.8 (9) Å^3^
*Z* = 8
Mo *K*α radiation
μ = 1.22 mm^−1^
*T* = 173 (2) K
0.42 × 0.22 × 0.07 mm

#### Data collection

Bruker APEXII CCD area-detector diffractometer
Absorption correction: integration (*XPREP*; Bruker, 2005 ▶) *T*_min_ = 0.750, *T*_max_ = 0.929
14324 measured reflections
3039 independent reflections
2214 reflections with *I* > 2σ(*I*)
*R*_int_ = 0.051

#### Refinement

*R*[*F* ^2^ > 2σ(*F* ^2^)] = 0.033
*wR*(*F* ^2^) = 0.084
*S* = 1.02
3039 reflections
164 parameters
H-atom parameters constrained
Δρ_max_ = 0.31 e Å^−3^
Δρ_min_ = −0.42 e Å^−3^

### 

Data collection: *APEX2* (Bruker, 2005 ▶); cell refinement: *SAINT-NT* (Bruker, 2005 ▶); data reduction: *SAINT-NT*; program(s) used to solve structure: *SHELXTL* (Sheldrick, 2008 ▶); program(s) used to refine structure: *SHELXTL*; molecular graphics: *ORTEP-3* (Farrugia, 1997 ▶); software used to prepare material for publication: *SHELXTL*.

## Supplementary Material

Crystal structure: contains datablocks global, I. DOI: 10.1107/S1600536808039524/dn2401sup1.cif

Structure factors: contains datablocks I. DOI: 10.1107/S1600536808039524/dn2401Isup2.hkl

Additional supplementary materials:  crystallographic information; 3D view; checkCIF report

## Figures and Tables

**Table 1:** Hydrogen-bond geometry (Å, °)

*D*—H⋯*A*	*D*—H	H⋯*A*	*D*⋯*A*	*D*—H⋯*A*
C1—H1⋯O1^i^	0.95	2.55	3.308 (3)	137
O1—H1*A*⋯*Cg*3^ii^	0.84	2.66	3.281 (2)	141
C2—H2⋯*Cg*1^iii^	0.95	2.90	3.766 (2)	155

Symmetry codes: (i) 
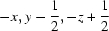
; (ii) 
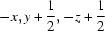
; (iii) 
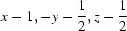
. *Cg*1 and *Cg*3 are the centroids of the unsubstituted Cp and the benzene rings, respectively.

**Fig. 1.**
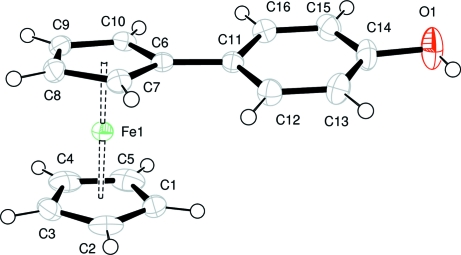
Molecular structure of the title complex with the atom labelling scheme. Ellipsoids are drawn at the 50% probability level.

## supplementary crystallographic information

### Comment

The synthesis of arylferrocenes especially compounds prepared by the reaction of *para*-substituted anilines *via* diazonium reactions to yield phenylferrocenes has evoked the interest of material scientists (Togni & Hayashi, 1995). For example, arylferrocenes have been established as precursors in the synthesis of ferrocenomesogens especially those with ferrocenyl moiety incorporated as a terminal group (Imrie *et al.*, 2002). These class of compounds are most readily prepared by cross-coupling reactions, *e.g.* of iodoferrocene (Imrie *et al.*, 2003) with arylboronic (Tsukazaki *et al.*, 1996) and organotin compounds (Lin *et al.*, 1995). Alternative cross-coupling reagents include aryl halides with tin (Guillaneux & Kagan, 1995), zinc (Foxman & Rosenblum, 1993) and ferrocenylboronic acids (Knapp & Rehahn, 1993). In this paper we report the synthesis of 4-hydroxyphenylferrocene using 4-aminophenol which was obtained *via* diazonium reaction.

The title compound (I) (Fig. 1) is a precursor prepared as part of a study to develop starting materials from cheaper sources for the development of new ionic liquid and liquid crystal materials (Nyamori & Bala, 2008*a*; 2008*b*). Due to the –OH subtituent on the benzyl ring it was thought that the property of (I) will be dominated by intra- or intermolecular hydrogen bonding, but analysis revealed no classical hydrogen bonds. Hence, the high melting point of 162 *^o^*C may be attributed to a concerted contribution from all molecular contacts within the crystal of (I) (Table 1: Cg(1) is the centroid of the unsubstituted Cp and Cg(3) the centroid of the benzene ring).

In the crystal of (I), the two cp rings are marginally tilted towards each other with a tilt angle between the planes of the two rings of 0.41 (5)°, while the interplanar angle between the cp and the phenyl ring is 13.0 (4)°.

### Experimental

In an excess of 2*M* hydrochloric acid at 5 °C was dissolved 4-aminophenol (12.00 g, 0.11 mol) followed by slow addition of sodium nitrite (8.00 g, 0.11 mol) in cold water (20 cm^3^) also at 5 °C. The solution was left to stir at this temperature for 30 min and the resultant solution was filtered. The filtrate was immediately added to a cold thoroughly stirred solution of ferrocene (18.00 g, 0.10 mol) in diethyl ether (500 cm^3^). Stirring was continued at 5 °C for 8 h. The ether layer was then separated, washed with water (3 *x* 100 cm^3^) and dried over anhydrous sodium sulfate. The solution was concentrated and the residue was passed through a column of alumina. Dichloromethane: hexane (1:1) eluted unreacted ferrocene. Further elution of the column with diethyl ether yielded 4-ferrocenylphenol (5.22 g, 32%) as yellow crystals recrystallized from hexane, mp 162 *^o^*C.

FTIR: *ν_max_*(KBr/cm^-1^) 3515, 3091, 1901, 1607, 1525, 1454, 1434, 1264, 1210, 1176, 1102, 1027, 998, 885, 839, 816, 665, 620; ^1^H-NMR: δ_H_(CDCl_3_) 7.38(2*H*, d, *J* 8.5, ArH), 6.79(2*H*, d, *J* 8.1, ArH), 4.87(1*H*, s,OH), 4.58(2*H*, t, *J* 1.9, C_5_H_4_), 4.28(2*H*, t, *J* 1.9, C_5_H_4_), 4.05(5*H*, s, C_5_H_5_); ^13^C-NMR: *δ_C_*(CDCl_3_) 154.25, 131.88, 127.76, 115.70, 86.192, 69.87, 68.89, 66.51; EI–MS 70 eV *m/z *(%): 280(39%), 277(*M*^+^, 81%), 276(100%), 220(10%), 213(39%); Found: *M*^+^, 278.0388 for C_16_H_14_FeO, requires *M*, 278.0392.

### Refinement

All H atoms attached to C atoms were fixed geometrically and treated as riding with C—H = 0.95 Å and *U*_iso_(H) = 1.2*U*_eq_(C). Hydrogen atom attached to oxygen was freely refined.

### Crystal data

**Table:**

[Fe(C_5_H_5_)(C_11_H_9_O)]	*F*_000_ = 1152
*M**_r_* = 278.12	*D*_x_ = 1.51 Mg m^−^^3^
Orthorhombic, *P**b**c**a*	Mo *K*α radiation λ = 0.71073 Å
Hall symbol: -P 2ac 2ab	Cell parameters from 946 reflections
*a* = 9.950 (2) Å	θ = 3.9–27.6º
*b* = 7.9205 (17) Å	µ = 1.22 mm^−^^1^
*c* = 31.046 (6) Å	*T* = 173 (2) K
*V* = 2446.8 (9) Å^3^	Plate, orange
*Z* = 8	0.42 × 0.22 × 0.07 mm

### Data collection

**Table:**

Bruker APEXII CCD area-detector diffractometer	*R*_int_ = 0.051
φ and ω scans	θ_max_ = 28.3º
Absorption correction: integration(XPREP; Bruker, 2005)	θ_min_ = 2.4º
*T*_min_ = 0.750, *T*_max_ = 0.929	*h* = −13→8
14324 measured reflections	*k* = −9→10
3039 independent reflections	*l* = −41→41
2214 reflections with *I* > 2σ(*I*)	

### Refinement

**Table:**

Refinement on *F*^2^	H-atom parameters constrained
Least-squares matrix: full	*w* = 1/[σ^2^(*F*_o_^2^) + (0.0424*P*)^2^ + 0.544*P*] where *P* = (*F*_o_^2^ + 2*F*_c_^2^)/3
*R*[*F*^2^ > 2σ(*F*^2^)] = 0.033	(Δ/σ)_max_ = 0.001
*wR*(*F*^2^) = 0.084	Δρ_max_ = 0.31 e Å^−^^3^
*S* = 1.02	Δρ_min_ = −0.42 e Å^−^^3^
3039 reflections	Extinction correction: none
164 parameters	

### Special details

**Table:**

Geometry. All e.s.d.'s (except the e.s.d. in the dihedral angle between two l.s. planes) are estimated using the full covariance matrix. The cell e.s.d.'s are taken into account individually in the estimation of e.s.d.'s in distances, angles and torsion angles; correlations between e.s.d.'s in cell parameters are only used when they are defined by crystal symmetry. An approximate (isotropic) treatment of cell e.s.d.'s is used for estimating e.s.d.'s involving l.s. planes.

### Fractional atomic coordinates and isotropic or equivalent isotropic displacement parameters (Å^2^)

**Table:**

	*x*	*y*	*z*	*U*_iso_*/*U*_eq_	
C1	−0.1342 (2)	0.1001 (3)	0.11907 (7)	0.0408 (6)	
H1	−0.1586	0.1489	0.1459	0.049*	
C2	−0.1895 (2)	0.1413 (3)	0.07829 (8)	0.0354 (5)	
H2	−0.2575	0.2228	0.0729	0.042*	
C3	−0.1253 (2)	0.0396 (3)	0.04727 (7)	0.0347 (5)	
H3	−0.1426	0.0401	0.0172	0.042*	
C4	−0.0312 (2)	−0.0627 (3)	0.06860 (9)	0.0380 (6)	
H4	0.0263	−0.1431	0.0554	0.046*	
C5	−0.0366 (2)	−0.0263 (3)	0.11265 (9)	0.0420 (6)	
H5	0.0163	−0.0778	0.1345	0.05*	
C6	0.10992 (19)	0.3807 (2)	0.10912 (6)	0.0210 (4)	
C7	0.0405 (2)	0.4386 (2)	0.07116 (6)	0.0250 (4)	
H7	−0.0281	0.522	0.0704	0.03*	
C8	0.0926 (2)	0.3492 (2)	0.03499 (6)	0.0263 (4)	
H8	0.065	0.3629	0.0059	0.032*	
C9	0.1930 (2)	0.2361 (3)	0.04994 (7)	0.0267 (4)	
H9	0.2441	0.1608	0.0326	0.032*	
C10	0.20381 (19)	0.2549 (2)	0.09535 (7)	0.0237 (4)	
H10	0.2634	0.1941	0.1135	0.028*	
C11	0.09297 (19)	0.4446 (2)	0.15359 (6)	0.0218 (4)	
C12	−0.0132 (2)	0.5527 (3)	0.16436 (7)	0.0281 (4)	
H12	−0.0789	0.5791	0.1432	0.034*	
C13	−0.0248 (2)	0.6219 (3)	0.20519 (7)	0.0332 (5)	
H13	−0.0975	0.6953	0.2117	0.04*	
C14	0.0701 (3)	0.5840 (3)	0.23658 (7)	0.0339 (5)	
C15	0.1749 (2)	0.4741 (3)	0.22686 (7)	0.0322 (5)	
H15	0.239	0.4458	0.2484	0.039*	
C16	0.1861 (2)	0.4061 (3)	0.18597 (6)	0.0266 (4)	
H16	0.2584	0.3317	0.1797	0.032*	
O1	0.0663 (2)	0.6509 (3)	0.27745 (5)	0.0519 (5)	
H1A	−0.0033	0.7099	0.2803	0.078*	
Fe1	0.01267 (3)	0.18508 (3)	0.078786 (8)	0.02002 (10)	

### Atomic displacement parameters (Å^2^)

**Table:**

	*U*^11^	*U*^22^	*U*^33^	*U*^12^	*U*^13^	*U*^23^
C1	0.0393 (14)	0.0524 (15)	0.0306 (12)	−0.0248 (12)	0.0100 (10)	−0.0062 (11)
C2	0.0181 (10)	0.0312 (11)	0.0567 (16)	−0.0031 (8)	−0.0022 (10)	0.0064 (10)
C3	0.0340 (12)	0.0417 (13)	0.0286 (11)	−0.0154 (10)	−0.0044 (9)	−0.0013 (10)
C4	0.0294 (12)	0.0196 (10)	0.0650 (17)	−0.0067 (9)	0.0036 (11)	−0.0031 (10)
C5	0.0360 (13)	0.0390 (14)	0.0511 (15)	−0.0137 (10)	−0.0123 (11)	0.0252 (12)
C6	0.0207 (10)	0.0187 (9)	0.0236 (10)	−0.0036 (7)	0.0012 (8)	0.0020 (7)
C7	0.0273 (11)	0.0198 (9)	0.0278 (11)	−0.0011 (8)	0.0005 (8)	0.0027 (8)
C8	0.0334 (12)	0.0248 (10)	0.0207 (10)	−0.0065 (8)	0.0018 (8)	0.0026 (8)
C9	0.0237 (10)	0.0268 (10)	0.0295 (11)	−0.0047 (8)	0.0069 (9)	−0.0064 (8)
C10	0.0192 (10)	0.0218 (10)	0.0303 (10)	−0.0020 (8)	−0.0016 (8)	−0.0009 (8)
C11	0.0234 (10)	0.0190 (9)	0.0230 (10)	−0.0025 (8)	0.0012 (8)	0.0009 (7)
C12	0.0285 (11)	0.0295 (11)	0.0262 (10)	0.0035 (9)	−0.0027 (8)	0.0009 (8)
C13	0.0378 (13)	0.0323 (11)	0.0294 (11)	0.0078 (10)	0.0053 (10)	−0.0026 (9)
C14	0.0487 (14)	0.0327 (12)	0.0202 (10)	−0.0010 (10)	0.0032 (10)	−0.0029 (8)
C15	0.0369 (12)	0.0348 (12)	0.0249 (11)	−0.0015 (10)	−0.0052 (9)	0.0032 (9)
C16	0.0265 (11)	0.0255 (11)	0.0278 (11)	0.0015 (8)	−0.0005 (9)	0.0025 (8)
O1	0.0721 (14)	0.0599 (12)	0.0237 (8)	0.0148 (10)	−0.0012 (9)	−0.0124 (8)
Fe1	0.01885 (16)	0.01960 (15)	0.02160 (15)	−0.00114 (11)	−0.00087 (11)	0.00129 (11)

### Geometric parameters (Å, °)

**Table:**

C1—C5	1.409 (4)	C7—H7	0.95
C1—C2	1.419 (3)	C8—C9	1.420 (3)
C1—Fe1	2.038 (2)	C8—Fe1	2.0425 (19)
C1—H1	0.95	C8—H8	0.95
C2—C3	1.409 (3)	C9—C10	1.422 (3)
C2—Fe1	2.041 (2)	C9—Fe1	2.046 (2)
C2—H2	0.95	C9—H9	0.95
C3—C4	1.404 (3)	C10—Fe1	2.046 (2)
C3—Fe1	2.042 (2)	C10—H10	0.95
C3—H3	0.95	C11—C12	1.400 (3)
C4—C5	1.399 (4)	C11—C16	1.401 (3)
C4—Fe1	2.036 (2)	C12—C13	1.386 (3)
C4—H4	0.95	C12—H12	0.95
C5—Fe1	2.037 (2)	C13—C14	1.390 (3)
C5—H5	0.95	C13—H13	0.95
C6—C10	1.431 (3)	C14—O1	1.376 (2)
C6—C7	1.441 (3)	C14—C15	1.392 (3)
C6—C11	1.480 (3)	C15—C16	1.384 (3)
C6—Fe1	2.0550 (19)	C15—H15	0.95
C7—C8	1.425 (3)	C16—H16	0.95
C7—Fe1	2.041 (2)	O1—H1A	0.84
			
C5—C1—C2	107.7 (2)	C16—C11—C6	121.30 (18)
C5—C1—Fe1	69.74 (13)	C13—C12—C11	121.55 (19)
C2—C1—Fe1	69.79 (12)	C13—C12—H12	119.2
C5—C1—H1	126.1	C11—C12—H12	119.2
C2—C1—H1	126.1	C12—C13—C14	119.9 (2)
Fe1—C1—H1	125.9	C12—C13—H13	120
C3—C2—C1	107.6 (2)	C14—C13—H13	120
C3—C2—Fe1	69.83 (12)	O1—C14—C13	123.0 (2)
C1—C2—Fe1	69.51 (12)	O1—C14—C15	117.5 (2)
C3—C2—H2	126.2	C13—C14—C15	119.50 (19)
C1—C2—H2	126.2	C16—C15—C14	120.2 (2)
Fe1—C2—H2	126	C16—C15—H15	119.9
C4—C3—C2	108.1 (2)	C14—C15—H15	119.9
C4—C3—Fe1	69.63 (12)	C15—C16—C11	121.37 (19)
C2—C3—Fe1	69.80 (12)	C15—C16—H16	119.3
C4—C3—H3	126	C11—C16—H16	119.3
C2—C3—H3	126	C14—O1—H1A	109.5
Fe1—C3—H3	126.2	C4—Fe1—C5	40.17 (10)
C5—C4—C3	108.5 (2)	C4—Fe1—C1	67.84 (10)
C5—C4—Fe1	69.96 (13)	C5—Fe1—C1	40.45 (10)
C3—C4—Fe1	70.11 (13)	C4—Fe1—C7	163.72 (9)
C5—C4—H4	125.8	C5—Fe1—C7	154.33 (10)
C3—C4—H4	125.8	C1—Fe1—C7	119.58 (10)
Fe1—C4—H4	125.7	C4—Fe1—C2	67.88 (9)
C4—C5—C1	108.1 (2)	C5—Fe1—C2	68.10 (9)
C4—C5—Fe1	69.86 (13)	C1—Fe1—C2	40.70 (9)
C1—C5—Fe1	69.81 (13)	C7—Fe1—C2	107.46 (9)
C4—C5—H5	125.9	C4—Fe1—C3	40.27 (9)
C1—C5—H5	125.9	C5—Fe1—C3	67.76 (10)
Fe1—C5—H5	126	C1—Fe1—C3	68.01 (9)
C10—C6—C7	106.89 (17)	C7—Fe1—C3	126.24 (9)
C10—C6—C11	126.23 (17)	C2—Fe1—C3	40.37 (9)
C7—C6—C11	126.81 (18)	C4—Fe1—C8	126.43 (10)
C10—C6—Fe1	69.26 (11)	C5—Fe1—C8	163.85 (10)
C7—C6—Fe1	68.89 (11)	C1—Fe1—C8	153.91 (10)
C11—C6—Fe1	129.17 (13)	C7—Fe1—C8	40.86 (8)
C8—C7—C6	108.17 (18)	C2—Fe1—C8	119.13 (9)
C8—C7—Fe1	69.62 (11)	C3—Fe1—C8	107.58 (9)
C6—C7—Fe1	69.92 (11)	C4—Fe1—C9	108.10 (9)
C8—C7—H7	125.9	C5—Fe1—C9	126.82 (10)
C6—C7—H7	125.9	C1—Fe1—C9	164.34 (10)
Fe1—C7—H7	126.1	C7—Fe1—C9	68.65 (8)
C9—C8—C7	108.17 (18)	C2—Fe1—C9	153.46 (9)
C9—C8—Fe1	69.80 (11)	C3—Fe1—C9	119.42 (9)
C7—C8—Fe1	69.52 (11)	C8—Fe1—C9	40.65 (8)
C9—C8—H8	125.9	C4—Fe1—C10	119.95 (9)
C7—C8—H8	125.9	C5—Fe1—C10	108.43 (9)
Fe1—C8—H8	126.3	C1—Fe1—C10	126.98 (9)
C8—C9—C10	108.12 (17)	C7—Fe1—C10	68.71 (8)
C8—C9—Fe1	69.55 (12)	C2—Fe1—C10	164.51 (9)
C10—C9—Fe1	69.69 (11)	C3—Fe1—C10	153.85 (9)
C8—C9—H9	125.9	C8—Fe1—C10	68.49 (8)
C10—C9—H9	125.9	C9—Fe1—C10	40.66 (8)
Fe1—C9—H9	126.4	C4—Fe1—C6	154.05 (9)
C9—C10—C6	108.65 (17)	C5—Fe1—C6	119.77 (9)
C9—C10—Fe1	69.65 (11)	C1—Fe1—C6	107.79 (9)
C6—C10—Fe1	69.90 (11)	C7—Fe1—C6	41.19 (8)
C9—C10—H10	125.7	C2—Fe1—C6	126.55 (9)
C6—C10—H10	125.7	C3—Fe1—C6	164.07 (9)
Fe1—C10—H10	126.4	C8—Fe1—C6	69.01 (8)
C12—C11—C16	117.44 (18)	C9—Fe1—C6	68.82 (8)
C12—C11—C6	121.19 (17)	C10—Fe1—C6	40.84 (8)
			
C5—C1—C2—C3	0.0 (2)	C6—C7—Fe1—C9	−81.82 (13)
Fe1—C1—C2—C3	59.70 (15)	C8—C7—Fe1—C10	81.31 (13)
C5—C1—C2—Fe1	−59.65 (15)	C6—C7—Fe1—C10	−38.02 (12)
C1—C2—C3—C4	−0.2 (2)	C8—C7—Fe1—C6	119.33 (18)
Fe1—C2—C3—C4	59.31 (15)	C3—C2—Fe1—C4	−37.48 (14)
C1—C2—C3—Fe1	−59.50 (15)	C1—C2—Fe1—C4	81.26 (16)
C2—C3—C4—C5	0.3 (2)	C3—C2—Fe1—C5	−80.96 (15)
Fe1—C3—C4—C5	59.67 (15)	C1—C2—Fe1—C5	37.78 (15)
C2—C3—C4—Fe1	−59.42 (15)	C3—C2—Fe1—C1	−118.7 (2)
C3—C4—C5—C1	−0.2 (2)	C3—C2—Fe1—C7	125.93 (14)
Fe1—C4—C5—C1	59.54 (15)	C1—C2—Fe1—C7	−115.34 (14)
C3—C4—C5—Fe1	−59.76 (15)	C1—C2—Fe1—C3	118.7 (2)
C2—C1—C5—C4	0.1 (2)	C3—C2—Fe1—C8	83.02 (15)
Fe1—C1—C5—C4	−59.58 (15)	C1—C2—Fe1—C8	−158.25 (14)
C2—C1—C5—Fe1	59.68 (15)	C3—C2—Fe1—C9	48.9 (2)
C10—C6—C7—C8	−0.3 (2)	C1—C2—Fe1—C9	167.63 (18)
C11—C6—C7—C8	176.84 (18)	C3—C2—Fe1—C10	−160.9 (3)
Fe1—C6—C7—C8	−59.33 (14)	C1—C2—Fe1—C10	−42.2 (4)
C10—C6—C7—Fe1	59.07 (13)	C3—C2—Fe1—C6	167.43 (13)
C11—C6—C7—Fe1	−123.83 (19)	C1—C2—Fe1—C6	−73.83 (16)
C6—C7—C8—C9	0.2 (2)	C2—C3—Fe1—C4	119.3 (2)
Fe1—C7—C8—C9	−59.30 (14)	C4—C3—Fe1—C5	−37.40 (14)
C6—C7—C8—Fe1	59.52 (14)	C2—C3—Fe1—C5	81.89 (15)
C7—C8—C9—C10	−0.1 (2)	C4—C3—Fe1—C1	−81.22 (16)
Fe1—C8—C9—C10	−59.22 (14)	C2—C3—Fe1—C1	38.08 (14)
C7—C8—C9—Fe1	59.13 (14)	C4—C3—Fe1—C7	167.43 (14)
C8—C9—C10—C6	−0.1 (2)	C2—C3—Fe1—C7	−73.28 (16)
Fe1—C9—C10—C6	−59.20 (13)	C4—C3—Fe1—C2	−119.3 (2)
C8—C9—C10—Fe1	59.13 (14)	C4—C3—Fe1—C8	126.14 (14)
C7—C6—C10—C9	0.2 (2)	C2—C3—Fe1—C8	−114.56 (14)
C11—C6—C10—C9	−176.92 (17)	C4—C3—Fe1—C9	83.45 (16)
Fe1—C6—C10—C9	59.04 (13)	C2—C3—Fe1—C9	−157.26 (13)
C7—C6—C10—Fe1	−58.84 (13)	C4—C3—Fe1—C10	49.3 (3)
C11—C6—C10—Fe1	124.04 (19)	C2—C3—Fe1—C10	168.58 (18)
C10—C6—C11—C12	−172.01 (19)	C4—C3—Fe1—C6	−158.9 (3)
C7—C6—C11—C12	11.4 (3)	C2—C3—Fe1—C6	−39.6 (4)
Fe1—C6—C11—C12	−80.3 (2)	C9—C8—Fe1—C4	−74.51 (15)
C10—C6—C11—C16	11.2 (3)	C7—C8—Fe1—C4	166.01 (12)
C7—C6—C11—C16	−165.41 (19)	C9—C8—Fe1—C5	−44.4 (4)
Fe1—C6—C11—C16	102.9 (2)	C7—C8—Fe1—C5	−163.9 (3)
C16—C11—C12—C13	1.3 (3)	C9—C8—Fe1—C1	169.29 (18)
C6—C11—C12—C13	−175.68 (19)	C7—C8—Fe1—C1	49.8 (2)
C11—C12—C13—C14	−0.3 (3)	C9—C8—Fe1—C7	119.48 (17)
C12—C13—C14—O1	178.6 (2)	C9—C8—Fe1—C2	−157.37 (12)
C12—C13—C14—C15	−1.1 (3)	C7—C8—Fe1—C2	83.15 (14)
O1—C14—C15—C16	−178.4 (2)	C9—C8—Fe1—C3	−114.96 (13)
C13—C14—C15—C16	1.3 (3)	C7—C8—Fe1—C3	125.56 (13)
C14—C15—C16—C11	−0.3 (3)	C7—C8—Fe1—C9	−119.48 (17)
C12—C11—C16—C15	−1.0 (3)	C9—C8—Fe1—C10	37.57 (12)
C6—C11—C16—C15	175.96 (19)	C7—C8—Fe1—C10	−81.91 (13)
C3—C4—Fe1—C5	119.37 (19)	C9—C8—Fe1—C6	81.54 (12)
C5—C4—Fe1—C1	−37.69 (14)	C7—C8—Fe1—C6	−37.94 (12)
C3—C4—Fe1—C1	81.68 (15)	C8—C9—Fe1—C4	125.35 (13)
C5—C4—Fe1—C7	−158.1 (3)	C10—C9—Fe1—C4	−115.18 (13)
C3—C4—Fe1—C7	−38.8 (4)	C8—C9—Fe1—C5	165.92 (13)
C5—C4—Fe1—C2	−81.80 (15)	C10—C9—Fe1—C5	−74.61 (15)
C3—C4—Fe1—C2	37.57 (13)	C8—C9—Fe1—C1	−162.4 (3)
C5—C4—Fe1—C3	−119.37 (19)	C10—C9—Fe1—C1	−42.9 (4)
C5—C4—Fe1—C8	167.52 (13)	C8—C9—Fe1—C7	−37.69 (12)
C3—C4—Fe1—C8	−73.11 (16)	C10—C9—Fe1—C7	81.77 (12)
C5—C4—Fe1—C9	126.18 (14)	C8—C9—Fe1—C2	48.8 (2)
C3—C4—Fe1—C9	−114.44 (14)	C10—C9—Fe1—C2	168.24 (17)
C5—C4—Fe1—C10	83.30 (15)	C8—C9—Fe1—C3	82.85 (14)
C3—C4—Fe1—C10	−157.33 (13)	C10—C9—Fe1—C3	−157.68 (12)
C5—C4—Fe1—C6	47.6 (3)	C10—C9—Fe1—C8	119.47 (16)
C3—C4—Fe1—C6	166.92 (17)	C8—C9—Fe1—C10	−119.47 (16)
C1—C5—Fe1—C4	−119.21 (19)	C8—C9—Fe1—C6	−82.04 (12)
C4—C5—Fe1—C1	119.21 (19)	C10—C9—Fe1—C6	37.43 (11)
C4—C5—Fe1—C7	166.05 (18)	C9—C10—Fe1—C4	83.08 (15)
C1—C5—Fe1—C7	46.8 (3)	C6—C10—Fe1—C4	−156.99 (13)
C4—C5—Fe1—C2	81.20 (14)	C9—C10—Fe1—C5	125.56 (14)
C1—C5—Fe1—C2	−38.01 (14)	C6—C10—Fe1—C5	−114.51 (14)
C4—C5—Fe1—C3	37.48 (13)	C9—C10—Fe1—C1	166.69 (13)
C1—C5—Fe1—C3	−81.73 (15)	C6—C10—Fe1—C1	−73.37 (15)
C4—C5—Fe1—C8	−38.7 (4)	C9—C10—Fe1—C7	−81.60 (13)
C1—C5—Fe1—C8	−157.9 (3)	C6—C10—Fe1—C7	38.34 (11)
C4—C5—Fe1—C9	−73.42 (16)	C9—C10—Fe1—C2	−160.1 (3)
C1—C5—Fe1—C9	167.37 (13)	C6—C10—Fe1—C2	−40.1 (4)
C4—C5—Fe1—C10	−114.89 (14)	C9—C10—Fe1—C3	48.6 (2)
C1—C5—Fe1—C10	125.90 (14)	C6—C10—Fe1—C3	168.58 (18)
C4—C5—Fe1—C6	−158.16 (13)	C9—C10—Fe1—C8	−37.57 (12)
C1—C5—Fe1—C6	82.62 (15)	C6—C10—Fe1—C8	82.37 (12)
C5—C1—Fe1—C4	37.44 (14)	C6—C10—Fe1—C9	119.93 (16)
C2—C1—Fe1—C4	−81.38 (15)	C9—C10—Fe1—C6	−119.93 (16)
C2—C1—Fe1—C5	−118.8 (2)	C10—C6—Fe1—C4	50.7 (2)
C5—C1—Fe1—C7	−158.69 (14)	C7—C6—Fe1—C4	169.36 (19)
C2—C1—Fe1—C7	82.48 (15)	C11—C6—Fe1—C4	−69.7 (3)
C5—C1—Fe1—C2	118.8 (2)	C10—C6—Fe1—C5	83.99 (15)
C5—C1—Fe1—C3	81.05 (15)	C7—C6—Fe1—C5	−157.38 (13)
C2—C1—Fe1—C3	−37.77 (14)	C11—C6—Fe1—C5	−36.5 (2)
C5—C1—Fe1—C8	166.23 (18)	C10—C6—Fe1—C1	126.50 (13)
C2—C1—Fe1—C8	47.4 (3)	C7—C6—Fe1—C1	−114.87 (13)
C5—C1—Fe1—C9	−40.4 (4)	C11—C6—Fe1—C1	6.1 (2)
C2—C1—Fe1—C9	−159.2 (3)	C10—C6—Fe1—C7	−118.63 (16)
C5—C1—Fe1—C10	−74.15 (17)	C11—C6—Fe1—C7	120.9 (2)
C2—C1—Fe1—C10	167.03 (12)	C10—C6—Fe1—C2	167.63 (13)
C5—C1—Fe1—C6	−115.30 (14)	C7—C6—Fe1—C2	−73.74 (15)
C2—C1—Fe1—C6	125.87 (14)	C11—C6—Fe1—C2	47.2 (2)
C8—C7—Fe1—C4	−43.9 (4)	C10—C6—Fe1—C3	−161.5 (3)
C6—C7—Fe1—C4	−163.3 (3)	C7—C6—Fe1—C3	−42.8 (3)
C8—C7—Fe1—C5	169.75 (19)	C11—C6—Fe1—C3	78.1 (4)
C6—C7—Fe1—C5	50.4 (3)	C10—C6—Fe1—C8	−80.98 (13)
C8—C7—Fe1—C1	−157.28 (13)	C7—C6—Fe1—C8	37.65 (12)
C6—C7—Fe1—C1	83.39 (14)	C11—C6—Fe1—C8	158.6 (2)
C8—C7—Fe1—C2	−114.61 (13)	C10—C6—Fe1—C9	−37.27 (12)
C6—C7—Fe1—C2	126.06 (13)	C7—C6—Fe1—C9	81.36 (13)
C8—C7—Fe1—C3	−74.04 (15)	C11—C6—Fe1—C9	−157.7 (2)
C6—C7—Fe1—C3	166.62 (12)	C7—C6—Fe1—C10	118.63 (16)
C6—C7—Fe1—C8	−119.33 (18)	C11—C6—Fe1—C10	−120.4 (2)
C8—C7—Fe1—C9	37.51 (12)		

### Hydrogen-bond geometry (Å, °)

**Table:**

*D*—H···*A*	*D*—H	H···*A*	*D*···*A*	*D*—H···*A*
C1—H1···O1^i^	0.95	2.55	3.308 (3)	137
O1—H1A···Cg3^ii^	0.84	2.66	3.281 (2)	141
C2—H2···Cg1^iii^	0.95	2.90	3.766 (2)	155

Symmetry codes: (i) −*x*, *y*−1/2, −*z*+1/2; (ii) −*x*, *y*+1/2, −*z*+1/2; (iii) *x*−1, −*y*−1/2, *z*−1/2.
